# Two *Nicotiana occidentalis* accessions enable gene identification for Type II hybrid lethality by the cross to *N. sylvestris*

**DOI:** 10.1038/s41598-021-96482-6

**Published:** 2021-08-24

**Authors:** Kenji Kawaguchi, Yuichiro Ohya, Maho Maekawa, Takahiro Iizuka, Akira Hasegawa, Kumpei Shiragaki, Hai He, Masayuki Oda, Toshinobu Morikawa, Shuji Yokoi, Takahiro Tezuka

**Affiliations:** 1 Graduate School of Life and Environmental Sciences, Osaka Prefecture University, Sakai, Osaka 599-8531 Japan; 2 School of Life and Environmental Sciences, Osaka Prefecture University, Sakai, Osaka 599-8531 Japan; 3 Education and Research Field, College of Life, Environment, and Advanced Sciences, Osaka Prefecture University, Sakai, Osaka 599-8531 Japan; 4 Bioeconomy Research Institute, Research Center for the 21St Century, Osaka Prefecture University, Sakai, Osaka 599-8531 Japan; 5 Present Address: NARO Hokkaido Agricultural Research Center, Memuro Research Station, 9-4 Shinsei-minami, Memuro, Kasai, Hokkaido 082-0081 Japan

**Keywords:** Plant biotechnology, Plant reproduction, Plant biotechnology, Plant breeding, Plant hybridization

## Abstract

Hybrid lethality, meaning the death of F_1_ hybrid seedlings, has been observed in many plant species, including *Nicotiana*. Previously, we have revealed that hybrids of the selected *Nicotiana occidentalis* accession and *N. tabacum*, an allotetraploid with S and T genomes, exhibited lethality characterized by the fading of shoot color. The lethality was suggested to be controlled by alleles of loci on the S and T genomes derived from *N. sylvestris* and *N. tomentosiformis*, respectively. Here, we extended the analysis of hybrid lethality using other two accessions of *N. occidentalis* identified from the five tested accessions. The two accessions were crossed with *N. tabacum* and its two progenitors, *N. sylvestris* and *N. tomentosiformis*. After crosses with *N. tabacum*, the two *N. occidentalis* accessions yielded inviable hybrid seedlings whose lethality was characterized by the fading of shoot color, but only the T genome of *N. tabacum* was responsible for hybrid lethality. Genetic analysis indicated that first-mentioned *N. occidentalis* accession carries a single gene causing hybrid lethality by allelic interaction with the S genome.

## Introduction

Wide hybridization, the creation of interspecific and intergeneric hybrids, is used by plant breeders to transfer desirable genes into domesticated species. However, attempts to produce wide hybrids are often hampered by pre and/or postzygotic barriers that reproductively isolates the species^1–3^. A prezygotic barrier prevents fertilization of the egg, while a postzygotic barrier prevents the formation of fertile offspring. Non-germination of pollen grains on the stigma and inhibition of pollen tube elongation are examples of the prezygotic barriers. Seed abortion, hybrid lethality and hybrid sterility in the F_1_ generation as well as hybrid breakdown in subsequent generations are examples of postzygotic barriers. Hybrid lethality has been reported in many systems including a great number of crop species^4–12^.

Hybrid lethality, which has been extensively studied in the genus *Nicotiana*, is as observed in *Nicotiana* interspecific hybrid seedlings and is classified into the five types based on the following early external symptoms: Type I, browning of the shoot apex and root tip; Type II, browning of the hypocotyl and roots; Type III, yellowing of true leaves; Type IV, formation of multiple shoots; and Type V, fading of shoot color^13,14^. Although several methods to overcome hybrid lethality have been reported in *Nicotiana*, their effectiveness is dependent on the type of hybrid lethality encountered. For example, Types I, II, III and V lethality are temperature sensitive; i.e., hybrid lethality is observed at 28 °C, but suppressed at elevated temperatures of approximately 34–36 °C. In contrast, Type IV lethality is not suppressed at elevated temperatures^5^. It is obvious that the lethality types might be determined by differences in genetic or allelic composition.

*Nicotiana tabacum* L., a commercially important cultivated tobacco species, is a natural allotetraploid (2n = 48, SSTT; section *Nicotiana*) that originated by interspecific hybridization of *N. sylvestris* Speg. & Comes (2n = 24, SS; section *Sylvestres*) with *N. tomentosiformis* Goodsp. (2n = 24, TT; section *Tomentosae*), along with chromosome doubling^15–18^. *Nicotiana* section *Suaveolentes* includes 26 species, most of which are endemic to Australasia, and the *Suaveolentes* species are geographically isolated from the majority of species in other *Nicotiana* sections, which are distributed in the Americas^17,18^. All species in section *Suaveolentes*, excluding exceptional lines or accessions, are allotetraploids and each species possesses 30–48 chromosomes. Section *Suaveolentes* is considered to have originated from a single polyploid event approximately six million years ago, followed by speciation^19,20^. Progenitors of this section have been estimated based on sequence analysis of three low-copy nuclear genes, nuclear ribosomal DNA, and regions of the plastid genome; the paternal is *N. sylvestris* and the maternal progenitor is a hybrid of species in sections *Petunioides* and *Noctiflorae*^20–23^.

Our previous studies indicated that after crosses with *N. tabacum*, 19 species in section *Suaveolentes* produce inviable hybrids showing Type II lethality^9,14,24^, whereas *N. occidentalis* H.-M. Wheeler (*a Suaveolentes* species) accession JT, supplied by Japan Tobacco Inc., yields inviable hybrids showing Type V lethality^25^. On the other hands, two *Suaveolentes* species, *N. benthamiana* Domin and *N. fragrans* Hooker, yield 100% viable hybrids^9,26^. Out of the first mentioned 19 species, 12 species have been crossed with *N. tabacum* Haplo-Q (2n = 47) or its F_1_ progeny (2n = 47) which are monosomic lines missing one of a pair of Q chromosomes (S-genome linkage group 11 in the *N. tabacum* linkage map^27,28^) in the S genome. These cross experiments revealed that hybrids possessing the Q chromosome are inviable while those missing the Q chromosome are viable with no lethal symptoms, and thus the Q chromosome encodes one or more genes leading to Type II lethality^9,24,28^. Recently, the causal gene at *N. tabacum Hybrid Lethality 1* (*NtHL1*) locus on the chromosome was identified as *Nitab4.5_0006549g0030.1*, which codes the coiled-coil, nucleotide-binding site and leucine-rich repeat class of resistance gene^29^. On the other hand, a segregation analysis identified a single dominant gene in *N. debneyi* Domin (section *Suaveolentes*); i.e., the *Hla1-1* allele of the *Hybrid Lethality A1* (*HLA1*) locus, triggering Type II lethality by interaction with allele(s) of gene(s), probably *NtHL1*, on the Q chromosome^26^. Because section *Suaveolentes* is a monophyletic group^17,20,30^ and Type II lethality caused by allelic interaction with gene(s) on the Q chromosome is widely observed in crosses between *Suaveolentes* species and *N. tabacum*, we considered that at least the above 12 species have the *Hla1-1* allele ^14,26^.

Crosses between *N. occidentalis* JT and the *N. tabacum* monosomic line for the Q chromosome gave different results from above mentioned cross experiments using 12 species: both hybrids possessing and missing the Q chromosome showed Type V lethality^25^. When two progenitors of *N. tabacum*, *N. sylvestris* and *N. tomentosiformis*, were crossed with *N. occidentalis* JT, each hybrid seedling showed Type II and Type V lethality, respectively. Based on the results, we inferred that although only the phenotype of Type V lethality is observed, Type II lethality is also functioning in the cross between *N. occidentalis* JT and *N. tabacum*^25^.

In the preliminary study, we have identified *N. occidentalis* among five accessions of the section *Suaveolentes* (PI 271991, PI 555541, PI 555687, PI 555689 and PI 555690) by flower morphology, flow cytometry, chromosome number, and molecular phylogenetic analyses based on internal transcribed spacer (ITS) region and simple sequence repeat (SSR) markers (Supplementary Note). In the present study, we extended the analysis of hybrid lethality in crosses between *N. occidentalis* and *N. tabacum* using the two accessions identified as *N. occidentalis*. The two accessions were crossed with *N. tabacum* and its two progenitors, *N. sylvestris* and *N. tomentosiformis*, to investigate whether the hybrid seedlings show hybrid lethality, and if so to determine the responsible genome(s). Furthermore, genetic analysis of Type II hybrid lethality gene(s) in *N. occidentalis* JT was conducted using the *N. occidentalis* accessions which were determined to yield inviable hybrid seedlings showing Type V lethality in the presence of the T genome, but yield viable hybrid seedlings in the absence of the T genome.

## Materials and methods

### Plant materials

*Nicotiana tabacum* (2n = 48, SSTT) ‘Red Russian’, *N. sylvestris* (2n = 24, SS), *N. tomentosiformis* (2n = 24, TT) and *N. occidentalis* JT accession (2n = 42) were used. Seeds for these plants were provided by the Leaf Tobacco Research Center, Japan Tobacco Inc. (Oyama, Japan). In addition, we used other two accessions of *N. occidentalis*, PI 555541 and PI 555690, provided by the United States *Nicotiana* Germplasm Collection^31^. All plants were cultivated in a greenhouse under natural day length.

### Intraspecific and interspecific crosses

Conventional crossing and sowing were carried out as follows: flowers of plants used as maternal parents were emasculated 1 day before anthesis and pollinated with the pollen of paternal parent plants. For interspecific crosses, *N. occidentalis* accessions were used as female parents, because in the previous study seeds were successfully obtained when *N. occidentalis* JT was used as the female parent in crosses with *N. tabacum* and its two progenitors, but the crosses in the opposite direction were unsuccessful using conventional cross-pollination^25^. We investigated the number of capsules obtained after crosses and seed germination rates to evaluate the presence or absence of reproductive barriers.

Seeds obtained were soaked in a 0.5% gibberellic acid (GA_3_) solution for 30 min and sterilized with 5% sodium hypochlorite for 15 min and washed with sterilized water in three times. The sterilized seeds were sown in Petri dishes containing 25 ml of 1/2 MS medium^32^ supplemented with 1% sucrose and solidified with 0.2% Gelrite (pH 5.8), and then cultured at 25 °C under continuous illumination (approximately 140 μmol m^−2^ s^−1^). Viable seedlings obtained from intraspecific and interspecific crosses were transplanted on a 3:1 mixture of peat moss (Super Cell Top V or Super Mix A; Sakata Seed Co., Yokohama, Japan) and vermiculite (Nittai Co., Osaka, Japan), and the plants were cultivated in a greenhouse.

### Chromosome analysis

To determine chromosome numbers, root tips were pretreated with distilled water for 24 h at 4 °C, followed by soaking in 2 mM 8-hydroxyquinoline for 4 h at 18 °C, and were then fixed in ethanol/acetic acid (3:1) overnight. The root tips were then hydrolyzed in 1 N HCl for 8 min at 60 °C, stained with Schiff’s reagent, and then squashed in 45% acetic acid. The number of chromosomes in two to four root tip cells for each plant was counted under a light microscope (BX50; Olympus, Tokyo, Japan). Three individuals were observed for each accession.

### RAPD analysis

Random amplified polymorphic DNA (RAPD) analysis was carried out as described by Williams et al.^33^ with some minor modifications as follows. Briefly, 20 random 10-mer oligonucleotide primers (Kit A) were obtained from Operon Technologies (Alameda, CA, USA). Reaction mixtures contained 20 mM Tris–HCl (pH 8.8), 10 mM KCl, 2 mM MgCl_2_, 10 mM (NH_4_)_2_SO_4_, 0.2 mM each dNTP, 0.5 µM primer, 20 ng template DNA, and 1.0 U Taq DNA polymerase (BioAcademia, Osaka, Japan) in a total volume of 20 µL. Polymerase chain reaction (PCR) amplification was performed using a PC-818 thermal cycler (Astec Corp.) programmed for 2 min at 94 °C for initial denaturation, followed by 45 cycles of 30 s at 94 °C, 30 s at 36 °C, 2 min at 72 °C, and a final extension of 5 min at 72 °C. PCR products were separated by electrophoresis in a 1.5% agarose gel in TBE buffer and stained with ethidium bromide to visualize DNA bands. During analysis, only intense and clear DNA bands were scored.

### Phenotypic analysis of populations segregating for hybrid lethality

Using PI 555541 and PI 555690, we estimated the number of hybrid lethality genes in the JT accession which shows hybrid lethality in the cross with *N. sylvestris*. Segregating populations for hybrid lethality were obtained after triple crosses where *N. occidentalis* F_1_ hybrids were crossed with *N. sylvestris* used as the paternal parent. In the populations, plants without and with browning of their hypocotyls and roots (hallmark symptoms of Type II lethality) were designated as ‘viable’ and ‘inviable’, respectively. Segregation of the viable and inviable plants were tested for goodness of fit to the expected ratio at the 5% significance level using the χ^2^ test.

## Results

### The type of hybrid lethality in crosses between two *N. occidentalis* accessions and *N. tabacum*

**Figure 1:**
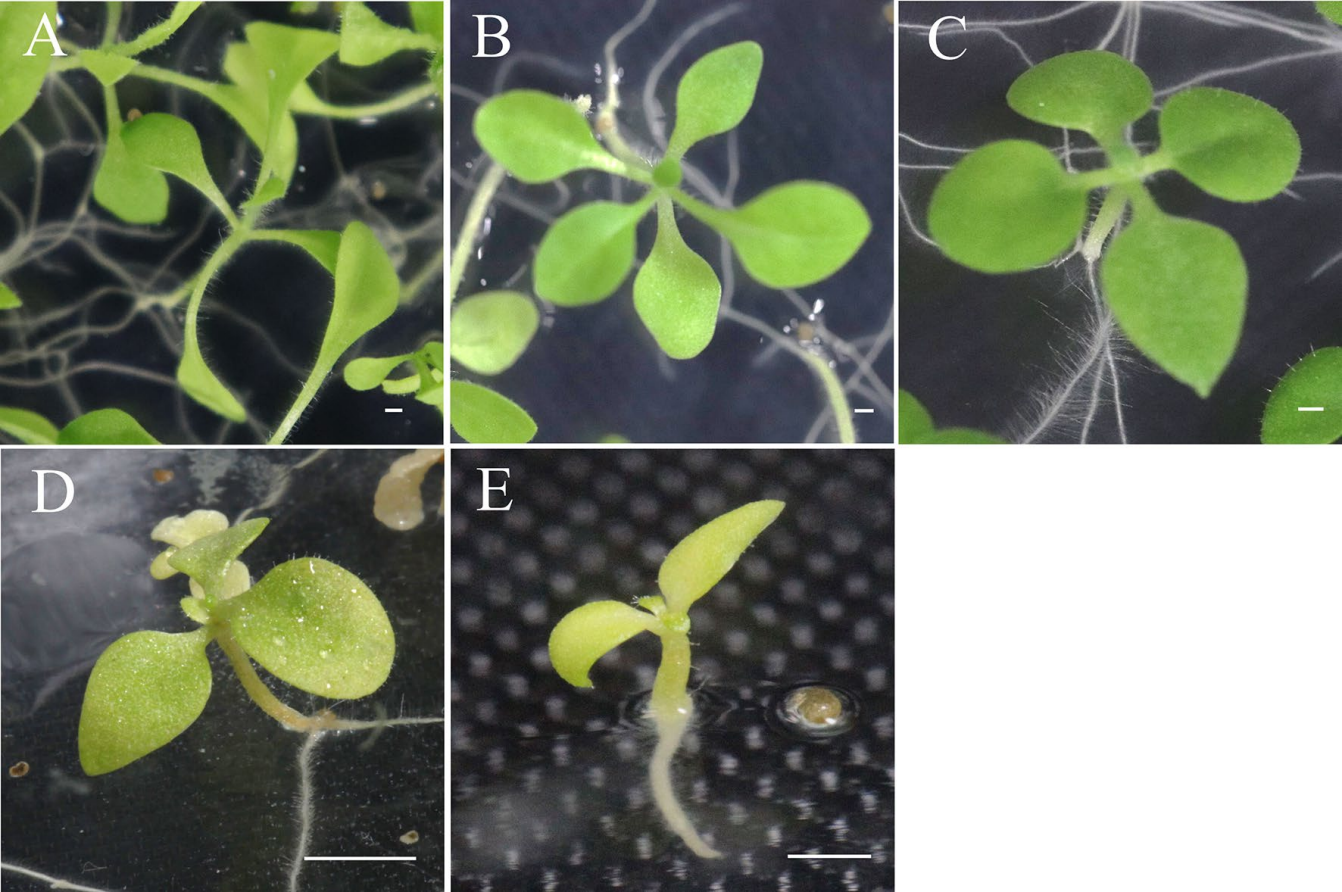
Observation of the characteristic early symptoms of hybrid lethality in hybrid seedlings between each accessions of *N. occidentalis* and *N. tabacum* at 25 °C. Fading of shoot color (Type V lethality) were observed. (**A**) PI 555541 at 10 DAG; (**B**) PI 555690 at 10 DAG; (**C**) *N. tabacum* ‘Red Russian’ at 10 DAG; (**D**) Hybrid between PI 555541 and *N. occidentalis* at 10 DAG; (**E**) Hybrid between PI 555690 and *N. occidentalis* at 10 DAG. Scale bar = 1 mm.

We conducted self-crosses of *N. occidentalis* PI 555541 and PI 555690 as controls, and interspecific crosses between the two accessions and *N. tabacum* (Table 1). In self-crosses, PI 555541 produced capsules at high rates (94%) but seed germination rate was 63%. Conversely, PI 555690 produced capsules at a rate of 58% but seed germination rate was high (96%). PI 555541 and PI 555690 yielded capsules and seeds at a rate of 100% after crosses with *N. tabacum*. However, seed germination rates were 42% in the cross PI 555541 × *N. tabacum* and 53% in the cross PI 555690 × *N. tabacum*. While the self-pollinated progenies of PI 555541 and PI 555690 showed no lethal symptoms, hybrid seedlings derived from crosses PI 555541 × *N. tabacum* and PI 555690 × *N. tabacum* showed fading of shoot color which is a typical symptom of Type V lethality (Table 1, Fig. 1).

**Table 1:** Efficiency of conventional crossings of *N. occidentalis* with *N. tabacum* and its two progenitors.

Cross combination	No. of flowers pollinated	No. of capsules obtained	No. of seeds sown	No. of plants or hybrids obtained			Lethality type
				Total	Viable	Inviable	
PI 555541 × *N. tabacum*	24	24 (100%^a^)	277	116 (42%^b^)	0	116	V
PI 555690 × *N. tabacum*	10	10 (100%)	393	209 (53%)	0	209	V
PI 555541 × *N. sylvestris*	4	3 (75%)	124	35 (28%)	35	0	–
PI 555541 × *N. tomentosiformis*	6	5 (83%)	344	123 (36%)	0	123	V
PI 555690 × *N. sylvestris*	9	8 (89%)	289	218 (75%)	218	0	–
PI 555690 × *N. tomentosiformis*	4	3 (75%)	396	42 (11%)	0	42	V
PI 555541	18	17 (94%)	231	146 (63%)	146	0	–
PI 555690	12	7 (58%)	334	321 (96%)	321	0	–

^a^Percentage of capsules obtained.

^b^Percentage of seed germination.

### The *N. tabacum* genome responsible for hybrid lethality in crosses between two *N. occidentalis* accessions and *N. tabacum*

To reveal *N. tabacum* genome responsible for hybrid lethality, two *N. occidentalis* accessions were crossed with two progenitors of *N. tabacum*, *N. sylvestris* and *N. tomentosiformis* (Table 1). PI 555541 yielded capsules and seeds at rates of 75% after the cross with *N. sylvestris* and 83% after the cross with *N. tomentosiformis*. Seed germination rates were comparatively low (28% in the cross with *N. sylvestris* and 36% in the cross with *N. tomentosiformis*). PI 555690 yielded capsules and seeds at rates of 89% after the cross with *N. sylvestris* and 75% after the cross with *N. tomentosiformis*. Seed germination rate was comparatively high in the cross with *N. sylvestris* (75%) but low in the cross with *N. tomentosiformis* (11%) (Table 1).

**Figure 2:**
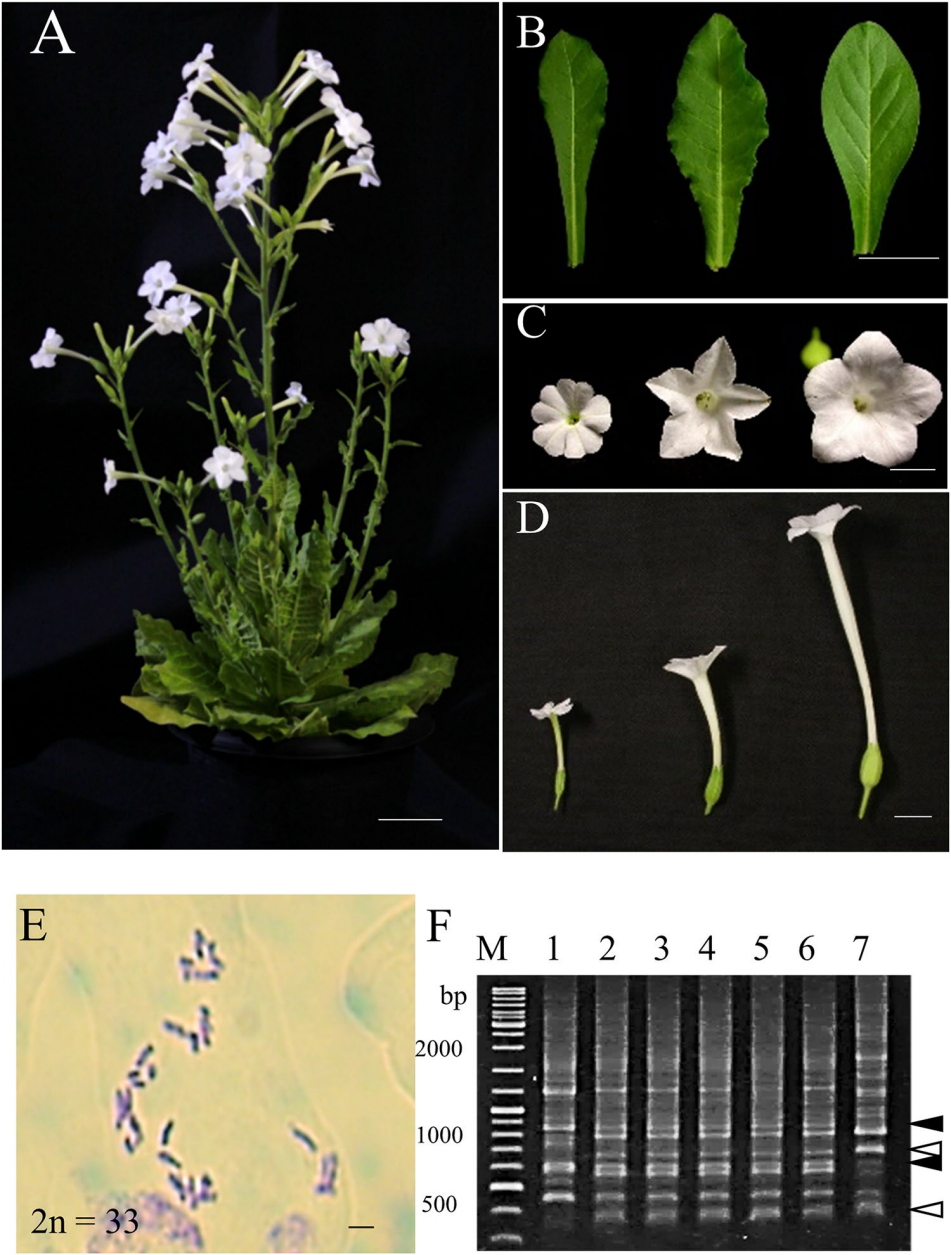
Hybrids from the cross *N. occidentalis* PI 555541 × *N. sylvestris*. (**A**) Shape of a hybrid plant that has grown to maturity and flowered. (**B**) Leaves of PI 555541, a hybrid plant and *N. sylvestris* (left to right). (**C**,**D**) Flowers of PI 555541, a hybrid plant and *N. sylvestris* (left to right). (**E**) Image of a root tip cell of hybrid plant showing the number of chromosomes. Scale bars = 10 cm (**A**), 5 cm (**B**), 1 cm (**C**,**D**) and 3 µm (**E**). (**F**) Confirmation of hybrid formation by RAPD analysis. M, DNA size marker (GeneRuler DNA ladder mix, Thermo fisher scientific, Waltham, USA). Lane 1, PI 555541; lanes 2–6, hybrid plants; lane 7, *N. sylvestris*. Both bands specific to PI 555541 (closed triangles) and those specific to *N. sylvestris* (open triangles) were detected in hybrids.

Hybrid seedlings from crosses PI 555541 × *N. tomentosiformis* and PI 555690 × *N. tomentosiformis* showed Type V lethality (Table 1). Conversely, seedlings from crosses PI 555541 × *N. sylvestris* (Fig. 2) and PI 555690 × *N. sylvestris* (Supplementary Fig. S12) grew to maturity and flowered. The seedlings from the cross PI 555541 × *N. sylvestris* were confirmed to be true hybrids: the mature seedlings displayed uniform morphological characteristics, with leaf and flower shapes that were intermediate in appearance between those of the parents (Fig. 2B–D). The chromosomal analysis of three seedlings randomly selected, revealed that each possessed 33 chromosomes, which is the sum of the number of haploid chromosomes of the parents (Fig. 2E). The five seedlings randomly selected were also subjected to RAPD analysis (Fig. 2F, Supplementary Fig. S11). Random primers gave RAPD patterns showing clear polymorphisms between the parents; 63 bands were detected only in PI 555541 and 57 bands were detected only in *N. sylvestris*. All seedlings had all 120 bands characteristic of both parents (Supplementary Table S4). Similarly, seedlings of the cross PI 555690 × *N. sylvestris* were confirmed to be true hybrids (Supplementary Table S4, Supplementary Figs. S12, and S13).

### Genetic analysis of *N. occidentalis* genes causing hybrid lethality by the interaction with the S genome

Previously, we have demonstrated that *N. occidentalis* JT yields inviable hybrids showing Type II and Type V lethality after crosses with *N. sylvestris* and *N. tomentosiformis*, respectively^25^. Meanwhile, *N. occidentalis* PI 555541 and PI 555690 yielded inviable hybrids showing Type V lethality only after crosses with *N. tomentosiformis*, and yielded viable hybrids after crosses with *N. sylvestris*. Therefore, using the two accessions identified in this study, it would be possible to genetically analyze Type II lethality observed in the cross *N. occidentalis* JT × *N. sylvestris*. To achieve this, we planned to obtain reciprocal hybrids between JT and PI 555541 or PI 555690, and then to cross the hybrids with *N. sylvestris*. After the triple crosses, the progeny should segregate into viable seedlings and inviable seedlings showing Type II lethality.

First, JT was reciprocally crossed with PI 555541 and PI 555690 (Table 2). Intraspecific hybrid seeds could be produced in all crosses. Among them, seed germination rate in the cross PI 555690 × JT was low (61%) compared with those in the other crosses (97–99%). Then, the hybrid plants were crossed with *N. sylvestris* to determine the segregation ratios of Type II lethality. After the triple crosses, seeds were obtained from all crosses, but the germination rates tended to be lower than those in self-crosses of *N. occidentalis* accessions and intraspecific hybrids (Table 2).

**Table 2:** Triple crosses between F_1_ hybrids of *N. occidentalis* accessions and *N. sylvestris.*

Cross combination	No. of flowers pollinated	No. of capsules obtained	No. of seeds sown	No. of hybrids			χ^2^ (1:1)	χ^2^ (3:1)
				Total	Viable	Inviable		
JT × PI 555541	28	16 (57%^a^)	151	150 (99%^b^)	150	0	–	–
PI 555541 × JT	26	18 (69%)	208	201 (97%)	201	0	–	–
JT × PI 555690	24	20 (83%)	123	120 (98%)	120	0	–	–
PI 555690 × JT	18	14 (78%)	90	55 (61%)	55	0	–	–
(JT × PI 555541) × *N. sylvestris*	51	22 (43%)	313	88 (28%)	53	35	3.6818	> 10*
(PI 555541 × JT) × *N. sylvestris*	47	31 (66%)	462	130 (28%)	80	50	6.9231*	> 10*
(JT × PI 555690) × *N. sylvestris*	49	24 (49%)	681	264 (39%)	130	134	0.0606	> 10*
(PI 555690 × JT) × *N. sylvestris*	15	7 (47%)	257	105 (41%)	54	51	0.0857	> 10*
Self-crossing of F_1_ (JT × PI 555690)	12	8 (67%)	173	137 (79%)	137	0	–	–

*Significantly different at 5% level from segregation model assuming one or two dominant genes.

^a^Percentage of capsules obtained.

^b^Percentage of seed germination.

Type II lethality in the cross JT × *N. sylvestris* was caused by epistatic interaction of dominant alleles, because the phenotype was observed in F_1_ hybrids. In many cases, hybrid lethality is genetically simple and caused by dominant alleles of two complementary genes^34–36^. Therefore, we assumed that JT possess a dominant allele for hybrid lethality locus and PI 555541 and PI 555690 possess a recessive allele at the locus. In the cross (JT × PI 555541) × *N. sylvestris*, the segregation ratio of viable and inviable seedlings was not significantly different from the expected 1:1 ratio for the monogenic inheritance. However, in the cross (PI 555541 × JT) × *N. sylvestris*, the segregation ratio was significantly different from the expected 1:1 ratio. Alternatively, we assumed that JT possess two dominant genes, and thus 3:1 ratio of viable to inviable seedlings. However, the segregation ratios did not fit the 3:1 ratio in both triple crosses (Table 2).

In crosses between reciprocal hybrids of JT × PI 55590 and *N. sylvestris*, the segregation ratios were not significantly different from 1:1 ratio at the 5% level. The hybrids of JT × PI 555690 cross were selfed and F_2_ plants were successfully obtained (Table 2).

## Discussion

Based on the results of crosses using two progenitors of *N. tabacum* (Table 1), we inferred the causal genome of *N. tabacum* for hybrid lethality in crosses between two *N. occidentalis* accessions and *N. tabacum*. The allele(s) in the T genome is obviously responsible for Type V lethality in crosses using PI 555541 and PI 555690. Meanwhile, in the cross between *N. occidentalis* JT and *N. tabacum*, hybrid lethality is controlled by alleles in both S and T genomes^25^. These phenotypic differences caused by the combination of alleles enabled genetic analysis of hybrid lethality in the cross *N. occidentalis* JT × *N. tabacum* or *N. occidentalis* JT × *N. sylvestris*.

Genetic analysis using triple crosses demonstrated that *N. occidentalis* JT has a single gene causing hybrid lethality by allelic interaction with the S genome of *N. tabacum* or *N. sylvestris*. In triple crosses including PI 555541, the number of viable seedlings tended to be larger than the number of inviable seedlings (Table 2). Although the χ^2^ values for 1:1 ratio differed between crosses (JT × PI 555541) × *N. sylvestris* and (PI 555541 × JT) × *N. sylvestris*, this difference might be caused by just numbers of individuals (large number tends to produce significant difference) and would be cross-direction independent in JT × PI 555541. Actually, frequencies of viable seedlings (or inviable seedlings) were similar between the two triple crosses. The germination rates of the F_1_ seeds obtained by crosses with *N. sylvestris* as male was 74.4% for JT^25^, 28.2% for PI 555541, and 75.4% for PI 555690. Several studies demonstrated that genes related to reproductive barriers, including hybrid lethality, hybrid sterility and gametophytic factors, cause segregation distortion^37–39^. If the JT allele causing hybrid lethality was linked in coupling phase with the possible JT gene related to seed germinability in the cross JT × *N. sylvestris*, the number of inviable seedlings is expected to be larger than the number of viable seedlings in triple crosses including PI 555541. This assumption conflicted with the crossing results. Alternatively, segregation distortion of hybrid lethality might occur solely due to the factors in PI 555541, which are related to low seed germinability after the cross with *N. sylvestris*. However, we could not exclude the possibility that genes causing hybrid lethality led to segregation distortion^38^.

We have previously estimated the evolutionary order and timing of causal genetic changes underlying hybrid lethality in the section *Suaveolentes* based on the phylogenetic tree^14,25^. In the models, the *Hla1-1* allele or other alleles of the *HLA1* locus, triggering Type II lethality by the interaction with the Q chromosome of *N. tabacum*, were acquired by diploid or allotetraploid ancestors of the section *Suaveolentes*, or by older species within the section *Suaveolentes*. Thus, many *Suaveolentes* species came to have the *Hla1-1* allele or the other alleles. Then, additional genetic changes reinforcing hybrid lethality accumulated in the lineage leading to *N. occidentalis* JT, giving rise to Type V lethality. Considering the results of the phylogenetic analysis and crossing experiments in the present study, loss of *Hla1-1* allele or another allele may have occurred in the lineage leading to *N. occidentalis* PI 555541 and PI 555690. On the other hand, loss of the *Hla1-1* allele or another allele as well as acquisition of factors triggering Type II lethality by the interaction with the T genome or genome of *N. tabacum* and *N. tomentosiformis* may have occurred in the lineage leading to *Nicotiana* sp. PI 555689 (Supplementary Note).

Further analysis using F_2_ plants derived from the cross *N. occidentalis* JT × PI555690 will reveal whether the hybrid lethality allele identified in JT in the present study is the *Hla1-1* allele at the *HLA1* locus or an allele at another locus, and will allow identification and cloning of the gene. A dual lethal system in the cross *N. occidentalis* JT × *N. tabacum* can provide a good model to study reinforcement of reproductive isolation.

## Supplementary Information

Supplementary Note.

Supplementary Figures.

Supplementary Tables.
