# Supplementary material for: Two Nicotiana occidentalis accessions enable gene identification for Type II hybrid lethality by the cross to N. sylvestris
Source: Sci Rep. 2021 Aug 24;11:17093. doi: 10.1038/s41598-021-96482-6 (PMC8384851; doi:10.1038/s41598-021-96482-6)
Supplement: Supplementary file 1 — Supplementary Note. [file 41598_2021_96482_MOESM1_ESM.pdf]

Supplementary Note

Two *Nicotiana occidentalis* accessions enable gene identification for Type II hybrid lethality by the cross to *N. sylvestris*

Kenji Kawaguchi[1,§], Yuichiro Ohya[1], Maho Maekawa[2], Takahiro Iizuka[1], Akira Hasegawa[2], Kumpei Shiragaki[1], Hai He[1], Masayuki Oda[1,3], Toshinobu Morikawa[1,3], Shuji Yokoi[1,3,4], Takahiro Tezuka[1,3,*]

[1]Graduate School of Life and Environmental Sciences, Osaka Prefecture University, Sakai, Osaka 599-8531, Japan
[2]School of Life and Environmental Sciences, Osaka Prefecture University, Sakai, Osaka 599-8531, Japan
[3]Education and Research Field, College of Life, Environment, and Advanced Sciences, Osaka Prefecture University, Sakai, Osaka 599-8531, Japan
[4]Bioeconomy Research Institute, Research Center for the 21st Century, Osaka Prefecture University, Sakai, Osaka 599-8531, Japan
[§]Present address: NARO Hokkaido Agricultural Research Center, Memuro Research Station, 9-4 Shinsei-minami, Memuro, Kasai, Hokkaido 082-0081, Japan

*Corresponding author: Takahiro Tezuka
Email: tezuka@plant.osakafu-u.ac.jp

Species confirmation of five accessions from the *Nicotiana* section *Suaveolentes*, and hybrid lethality in crosses using the identified non-*N. occidentalis* accessions

To identify *N. occidentalis* among five accessions of the section *Suaveolentes* (PI 271991, PI 555541, PI 555687, PI 555689 and PI 555690), we conducted morphological analysis, flow cytometry, chromosome analysis, and molecular phylogenetic analyses based on internal transcribed spacer (ITS) region and simple sequence repeat (SSR) markers. Subsequently, we crossed three accessions of identified non-*N. occidentalis* with *N. tabacum* and its two progenitors to obtain further insight into *Nicotiana* hybrid lethality.

Materials and Methods

Plant materials. Five accessions of the section *Suaveolentes*, PI 271991, PI 555541, PI 555687, PI 555689 and PI 555690, were used. These plants were provided as *N. occidentalis* by the United States *Nicotiana* Germplasm Collection[1]. Additionally, we used *N. occidentalis* JT accession (2n = 42) provided by the Leaf Tobacco Research Center, Japan Tobacco Inc. (Oyama, Japan). *N. occidentalis* can be divided into three subspecies, i.e., ssp. *occidentalis*, ssp. *obliqua* and ssp. *hesperis*[2]. PI 271991 and PI 555687 were provided as ssp. *hesperis* (also known as *N. hesperis*). No subspecies information was available for JT, PI 555541 and PI 555690. We also used *N. tabacum* (2n = 48, SSTT) 'Red Russian', *N. sylvestris* (2n = 24, SS), and *N. tomentosiformis* (2n = 24, TT), which were provided by the Leaf Tobacco Research Center. All plants excluding those used for morphological analysis were cultivated in a greenhouse under natural day length. For morphological analysis, plants were grown in an incubator under conditions of 16 hours light phase (140 μmol m^{-2} s^{-1}) and 8 hours dark phase at 25 °C.

Morphological analysis. We visually evaluated the color and shape of the flower in five *Suaveolentes* accessions and *N. occidentalis* JT. In addition, corolla tube length and corolla limb width were examined with calipers when the flowers fully opened. In these investigations, three to four plants of each accession were grown in separate plastic pots (13 cm diameter, 12 cm depth; soil volume approximately 1.2 L) filled with a 3:1 mixture of peat moss (Super Cell Top V or Super Mix A; Sakata Seed Co., Yokohama, Japan) and

vermiculite (Nittai Co., Osaka, Japan). Five flowers per plant were recorded for each characteristic. The corolla tube length and corolla limb width among the six accessions were compared by Tukey-Kramer multiple comparison test.

Flow cytometry. Five *Suaveolentes* accessions were analyzed by flow cytometry using external standardization[3,4] where JT was used as the standard and then each sample was analyzed. For flow cytometric analysis, nuclei were isolated from mature leaves of five *Suaveolentes* accessions and *N. occidentalis* JT. The leaves were chopped and macerated in ice-cold Otto I buffer[5]. The solution was filtered through 30 μm nylon mesh. Otto II buffer[3] supplemented with 4′,6-diamidino-2-phenylindole (DAPI; final concentration 5μl ml^{-1}) was added to the solution. The DNA content of the isolated nuclei was analyzed by a flow cytometer CyFlow Space (Partec GmbH, Münster, Germany). At least 10,000 nuclei were counted for each sample. Based on the histograms obtained by flow cytometry, DNA ploidy was estimated by WinMDI 2.9 software (http://www.cyto.purdue.edu/flowcyt/software/Winmdi.htm). Four plants were analyzed for each accession.

Construction of phylogenetic tree using the ITS region. Total DNA was extracted from the leaves of each plant using the cetyltrimethylammonium bromide method[6]. The ITS DNA region was amplified by conventional PCR using the primers described by Baldwin[7]. A 2.0–4.0% solution of DMSO was added to PCR reaction mixtures, because GC-rich regions in the ITS sequences are reported to cause premature termination of most strands within 100–150 bp of the initiation point[8]. The PCR was performed using a PC-818 thermal cycler (Astec Corp., Kasuya, Japan) programmed for 2 min at 94 °C for initial denaturation, followed by 28 cycles of 94°C for 30 sec, 55°C for 1 min and 72°C for 1 min, and final extension of 3 min at 72°C. The amplified DNA fragment was purified using Plus Gel Elution Kit (GMbiolab, Dali City, Taiwan) according to the manufacturer’s protocols. Purified PCR products were cloned into the T-vector (pBluescript II SK (+); Agilent Technologies, Inc., California, USA) and the ligated product was transformed into *Escherichia coli* DH5α competent cell. The purified DNA was sequenced using an ABI 3130xl automated sequencer (Applied Biosystems Inc., Warrington, UK) using standard fluorescent dye-terminator chemistry according to the

manufacturer's protocols. Sequences was determined for both DNA strands, and each base position was individually examined for agreement of the two strands. DNA sequences were deposited to the DNA Data Bank of Japan (accession numbers LC479090 to LC479096). We downloaded ITS sequences of 21 species (total 29 accessions) belonging to section *Suaveolentes*, *N. tabacum*, and two species from other genera of Solanaceae for use as an outgroup from the NCBI-Genbank database. The retrieved sequences were saved and fasta formatted for multiple sequence alignment. In total, 39 sequences were aligned using CLUSTAL W[9] with default setting. The aligned file was exported for phylogenetic analysis. Alignment gaps were treated as missing data. The maximum likelihood (ML) method was used to perform phylogenetic analysis and was performed with Kimura-2-parameter-gammadistance[10] proposed as the best-fit substitution model using MEGA 7 software[11]. In the phylogenetic tree, bootstrap percentages (BP) were calculated with 1000 replicates[12].

Construction of phylogenetic tree using SSR markers. SSR analysis was carried out using 19 primer sets described by Bindler et al.[13] (Supplementary Table S1). Reaction mixtures contained 10× ThermoPol Reaction Buffer (New England Biolabs, Massachusetts, USA), 0.2 mM each dNTP, 0.2 μM of each primer, 20 ng template DNA, and 0.5 U Taq DNA polymerase (BioAcademia, Osaka, Japan) in a total volume of 20 μl. PCR amplification was performed using a PC-818 thermal cycler (Astec Corp.) programmed for 1 min at 94°C for initial denaturation, followed by 35 cycles of 30 s at 94°C, 1 min at the appropriate temperature for each primer set and 1 min at 72°C, and a final extension of 3 min at 72°C. PCR products were separated by electrophoresis in a 3% agarose gel in TBE buffer and stained with ethidium bromide to visualize DNA bands. During analysis, only intense and clear DNA bands were scored, and the data matrix was generated based on the individual bands. The polymorphism information content (PIC) values were computed using the formula of Botstein et al.[14]. Phylogenetic analysis based on the SSR markers dataset was conducted by the maximum parsimony (MP) method using MEGA 7[11]. The branch-swapping algorithm (tree-bisection-reconnection; TBR) was implemented to search of phylogenetic trees[15]. In the phylogenetic tree, BP was calculated with 1000 replicates[12]. Consistency index (CI), retention index (RI), and composite index (CPI) were calculated by MEGA 7. The closer these values are to 1, the

closer they are to the phylogenetic tree obtained from the theoretical tree.

Results and Discussion

Species confirmation of *Suaveolentes* accessions based on flower morphology, chromosome number, and molecular phylogenetic analysis. We characterized the *Suaveolentes* accessions by flower morphology, ploidy level, chromosome number and molecular phylogenetic analysis. The flower morphology of five accessions and *N. occidentalis* JT is shown in Supplementary Fig. S1. The corolla limbs were white and corolla tubes were yellowish green in all accessions. Although corolla lobe shapes were emarginate in all accessions, only the corolla lobes of PI 555689 were deeply incised and had a star-like shape, whereas the other five accessions were slightly incised and showed shapes close to circular (Fig. S1A-B). When corolla tube lengths were compared, PI 555689 was the longest and PI 271991 was the second longest. Corolla tube lengths were the same between PI 555541 and PI 555690, and between JT and PI 555687 (Fig. S1C). A similar pattern was observed for corolla limb width (Fig. S1D). Although not statistically significant, the width of JT tended to be slightly longer than PI 555687 (Fig. S1D).

Flow cytometry and chromosome analysis were performed to determine ploidy levels and chromosome numbers in the accessions, respectively. Flow cytometry revealed that all accessions except PI 271991 showed the same fluorescence intensity. On the other hand, the G_1 peak of PI 271991 exhibited approximately twice the fluorescence intensity value compared to the other five accessions, suggesting the higher ploidy level (Supplementary Fig. S2). This was confirmed by chromosome analysis, conducted as described in the main article (Supplementary Fig. S3). PI 555541 and PI 555690 had 42 chromosomes which is the same number as in JT[16]. On the other hand, PI 271991, PI 555687, PI 555689 had 62, 32 and 40 chromosomes, respectively.

We performed molecular phylogenetic analysis based on ITS region and SSR markers to confirm the species of the *Suaveolentes* accessions. In molecular phylogenetic analysis of the ITS region, a single band was obtained in each of the accessions after PCR reaction. A single sequence was obtained in each of five accessions, whereas two sequences were obtained from PI 271991. The latter two sequences were named PI 271991_A and PI 271991_B. Among the ITS sequences (589 bp) of *Suaveolentes* species

used to construct phylogenetic tree, 39 bp variable sites were present. In the phylogenetic tree (Supplementary Fig. S4), two accessions, PI 555541 and PI 555690, were closely related to JT and *N. occidentalis* (ITS accession number AJ492417), and formed a strongly supported clade with BP of 99%. On the other hand, three accessions, PI 271991, PI 555687 and PI 555689, were included in different clades. Both PI 271991_A and PI 271991_B were located in a clade including *N. suaveolens* octoploid accessions[17] (BP = 51, 62). PI 555687 was located in a clade containing *N. suaveolens* with the same chromosome number as PI 555687 (BP < 50). PI 555689 was located in the clade containing species with large flowers such as *N. megalosiphon* (BP = 58) and *N. simulans* (BP < 50); both species have the same chromosome number as PI 555689[16].

In analysis using 19 SSR markers, a total of 62 alleles (2–4 alleles per marker) were detected among six certain or possible *N. occidentalis* accessions and *N. tabacum* (Supplementary Table S1). The phylogenetic tree based on SSR markers are shown in Supplementary Fig. S5. The CI is 0.8530, the RI is 0.6667, and the CPI is 0.5926 for all sites and parsimony-informative sites. In the phylogenetic tree, JT, PI 555541 and PI 555690 formed a clade. Although this was consistent with the results using ITS region, JT was located nearby PI 555541 and was somewhat distantly related with PI 555690 in the tree based on SSR markers. PI 271991 and PI 555689 formed a clade (BP = 67), and PI 555687 was located in another clade.

Based on these results, we determined that PI 555541 and PI 555690 as well as JT were *N. occidentalis*, while PI 271991, PI 555687, and PI 555689 were other *Nicotiana* species. *N. occidentalis* may be classified into three subspecies: ssp. *occidentalis* (corolla tube length is about 3.4 to 4.8 cm), ssp. *obliqua* (corolla tube length is about 1.5 to 3.6 cm), and ssp. *hesperis* (corolla tube length is about 1.1 to 1.8 cm)[2]. When judged by corolla tube length, *N. occidentalis* JT might correspond to ssp. *obliqua* or ssp. *hesperis*, and PI 555541 and PI 555690 might correspond to ssp. *obliqua* (Supplementary Fig. S1).

Among three non-*N. occidentalis* accessions, PI 271991 had 62 chromosomes and is considered to be octoploid (Supplementary Fig. S2, Supplementary Fig. S3). This accession was the only one that had two ITS sequences. Most diploid and tetraploid plant species, including those in the genus *Nicotiana*, have a single ITS sequence as a result of rapid concerted evolution[18]. On the other hand, paralogous ITS sequences have been confirmed in *Nicotiana* section *Alatae*[19]. Therefore, it is possible that the paralogs were

detected in PI 271991. In any case, these two ITS sequences were located in the same clade that includes *N. suaveolens* octoploid accessions[17] (Supplementary Fig. S4). Although BP values were 62 or 51, PI 271991 and the *N. suaveolens* accessions were similar in flower size and chromosome number[17]. Furthermore, Marks et al.[20] reported a presumable *N. suaveolens* accession with 62 chromosomes. Therefore, PI 271991 might be octoploid *N. suaveolens*.

Phylogenetic analysis of PI 555687 using ITS region showed that this accession was located on a clade containing *N. suaveolens* and *N. amplexicaulis* accessions (Supplementary Fig. S4). PI 555687 had 32 chromosomes (Supplementary Fig. S3), which is identical to the chromosome number of tetraploid *N. suaveolens*. Nevertheless, it is insufficient for species identification because the BP was low in the ITS phylogenetic tree. In addition, the length of corolla tube and corolla limb was also significantly smaller than *N. suaveolens* reported by Horton[2] and He et al.[17].

In the ITS phylogenetic tree, PI 555689 was located close to *N. megalosiphon* and *N. simulans* (Supplementary Fig. S4). The star-like shape flower of these two species were consistent with the features of PI 555689. Furthermore, PI 555689 had 40 chromosomes (Supplementary Fig. S3), which is identical to the chromosome number of *N. megalosiphon* and *N. simulans*. Therefore, this accession is likely to be either *N. megalosiphon* or *N. simulans*; the corolla length is consistent with *N. megalosiphon*, and both species are closely related in the ITS phylogenetic tree (Supplementary Fig. S4).

Hybrid lethality in interspecific crosses using three accessions of non-*N. occidentalis*. The results of self-crosses and crosses between three *Suaveolentes* accessions and *N. tabacum* are shown in Supplementary Table S2. In self-crosses, the accessions produced capsules at a rate of 50–70% but seed germination rates were high (≥ 88%). In crosses with *N. tabacum*, all accessions yielded capsules and seeds at high rates (≥ 80%). Seeds derived from the crosses using PI 555687 germinated well (92%). On the other hand, seed germination rates in the cross using PI 555689 was low (16%). Because empty seeds were obtained and none of the seeds germinated in the cross PI 271991 × *N. tabacum*, we conducted the reciprocal cross. Although two capsules were obtained, these capsules did not enlarge and contained powdery seeds. To obtain hybrid seedlings between PI 271991 and *N. tabacum*, ovule culture was carried out (Supplementary Table S3). As a result, only

one hybrid seedling could be obtained from ovules cultured at 8 days after pollination. Hybrid seedlings obtained by crosses using PI 271991, PI 555687 and PI 555689 showed browning of hypocotyls as an early symptom, a typical of Type II lethality (Supplementary Table S2, Supplementary Fig. S6D-F).

To reveal *N. tabacum* genome responsible for hybrid lethality, three *Suaveolentes* accessions were crossed with two progenitors of *N. tabacum*, *N. sylvestris* and *N. tomentosiformis* (Supplementary Table S2). Because crosses between *Suaveolentes* species and *N. tabacum* progenitors using conventional cross-pollination are generally successful when *Suaveolentes* species are used as female parents[21,22], we first crossed three *Suaveolentes* accessions as female parents with *N. tabacum* progenitors. In cases where seeds could not be obtained in the crosses, we conducted the reciprocal crosses where *Suaveolentes* accessions were used as male parents. As summarized in Supplementary Table S2, four crosses produced no capsules because flowers dropped with no ovary enlargement within 7 days after pollination. In crosses between PI 271991 as female parents and *N. sylvestris* or *N. tomentosiformis*, ovaries enlarged after pollination but the most of those dropped at approximately 7 days after pollination. Similar situations have been observed in crosses between *N. suaveolens* accessions (8x or 16x) and *N. tabacum* (4x)[17,22]. The ovary abscission is considered to be due to the maternal genome or effective ploidy excess compared with the paternal one[22]. Only two capsules were obtained in the cross PI 271991 × *N. tomentosiformis* but contained empty seeds which did not germinate. The other three crosses produced some germinable seeds.

Hybrid seedlings from the cross PI 555687 × *N. tomentosiformis* grew to maturity and flowered (Supplementary Fig. S7). The seedlings were confirmed to be true hybrids based on flower shape, chromosome number, and RAPD patterns conducted as described in the main article (Supplementary Table S4, Supplementary Fig. S7, Supplementary Fig. S8). Therefore, the S genome is likely to be involved in Type II lethality in the cross between PI 555687 and *N. tabacum*; however, direct proof could not be obtained.

Hybrid seedlings from the cross PI 555689 × *N. tomentosiformis* showed Type II lethality, suggesting that the T genome is involved in Type II lethality in the cross between PI 555689 and *N. tabacum* (Supplementary Table S2). Seedlings from the cross PI 555689 × *N. sylvestris* grew to maturity and flowered, but were not hybrids (Supplementary Table S4, Supplementary Fig. S9, Supplementary Fig. S10).

Our previous studies revealed that Type II lethality, where only the Q chromosome in the S genome is involved as the *N. tabacum* side factor, is commonly and widely observed in crosses between the *Suaveolentes* species and *N. tabacum*[21,23-25]. This fact might be applicable to *Nicotiana* sp. PI 555687. Meanwhile, hybrid lethality observed in the cross using PI 555689 is the first case of T genome-controlling Type II lethality in crosses between the *Suaveolentes* species and *N. tabacum*.

References

1. Lewis, R. S. & Nicholson, J. S. Aspects of the evolution of *Nicotiana tabacum* L. and the status of the United States *Nicotiana* Germplasm Collection. *Genet. Resour. Crop Evol.* **54**, 727–740 (2007).
2. Horton, P. A taxonomic revision of *Nicotiana* (Solanaceae) in Australia. *J. Adel. Bot. Gard.* **3**, 1–56 (1981).
3. Hendrix, B. & Stewart, J. M. Estimation of the nuclear DNA content of *Gossypium* species. *Ann. Bot.* **95**, 789–797 (2005).
4. Doležel, J., Greilhuber, J. & Suda, J. Estimation of nuclear DNA content in plants using flow cytometry. *Nat. Protoc.* **2**, 2233–2244 (2007).
5. Otto, F. DAPI staining of fixed cells for high-resolution flow cytometry of nuclear DNA. *Methods in Cell Biol.* **33**, 105–110 (1990).
6. Murray, M. G. & Thompson, W. F. Rapid isolation of high molecular weight plant DNA. *Nucleic Acids Res*. **8**, 4321–4325 (1980).
7. Baldwin, B. G. Phylogenetic utility of the internal transcribed spacers of nuclear ribosomal DNA in plants: an example from the Compositae. *Mol. Phylogenet. Evol.* **1**, 3–16 (1992).
8. Chase, M. W. *et al.* Molecular systematics, GISH and the origin of hybrid taxa in *Nicotiana* (Solanaceae). *Ann. Bot.* **92**, 107–127 (2003).
9. Thompson, J. D., Higgins, D. G. & Gibson, T. J. CLUSTAL W: improving the sensitivity of progressive multiple sequence alignment through sequence weighting, positions-specific gap penalties and weight matrix choice. *Nucleic Acids Res.* **22**, 4673–4680 (1994).
10. Kimura, M. A. simple method for estimating evolutionary rate of base substitutions through comparative studies of nucleotide sequences. *J. Mol. Evol.* **16**, 111–120

(1980).

11. Kumar, S., Stecher, G. & Tamura, K. MEGA7: Molecular Evolutionary Genetics Analysis Version 7.0 for Bigger Datasets. *Mol. Biol. Evol.* **33**, 1870–1874 (2016).
12. Felsenstein, J. Confidence limits on phylogenies: an approach using the bootstrap. *Evolution* **39**, 783–791 (1985).
13. Bindler, G. *et al*. A high density genetic map of tobacco (*Nicotiana tabacum* L.) obtained from large scale microsatellite marker development. *Theor. Appl. Genet.* **123,** 219–230 (2011).
14. Botstein, D., White, R. L., Skolnick, M. & Davis, R. W. Construction of a genetic linkage map in man using restriction fragment length polymorphisms. *Am. J. Hum. Genet.* **32**, 314–331 (1980).
15. Nei, M. & Kumar, S. Molecular Evolution and Phylogenetics (ed. Nei, M.) (Oxford University Press, 2000).
16. Japan Tobacco Inc. *The genus Nicotiana illustrated*. (Seibundo Shinkosha, 1994).
17. He, H. *et al*. *Nicotiana suaveolens* accessions with different ploidy levels exhibit different reproductive isolation mechanisms in interspecific crosses with *Nicotiana tabacum*. *J. Plant Res.* **132**, 461–471 (2019).
18. Lim, K. Y., Matyášek, R., Lichtenstein, C. P. & Leitch, A. R. Molecular cytogenetic analyses and phylogenetic studies in the *Nicotiana* section Tomentosae. *Chromosoma* **109**, 245–258 (2000).
19. Buckler, E. S., Ippolito, A. & Holtsford, T. P. The evolution of ribosomal DNA: Divergent paralogues and phylogenetic implications. *Genetics* **145**, 821–832 (1997).
20. Marks, C. E., Newbigin, E. & Ladiges, P. Y. Comparative morphology and phylogeny of *Nicotiana* section *Suaveolentes* (Solanaceae) in Australia and the South Pacific. *Aust. Syst. Bot.* **24**, 61–86 (2011).
21. Tezuka, T., Kuboyama, T., Matsuda, T. & Marubashi, W. Possible involvement of genes on the Q chromosome of *Nicotiana tabacum* in expression of hybrid lethality and programmed cell death during interspecific hybridization to *Nicotiana debneyi*. *Planta* **226**, 753–764 (2007).
22. He, H., Yokoi, S. & Tezuka, T. A high maternal genome excess causes severe seed abortion leading to ovary abscission in *Nicotiana* interploidy-interspecific crosses. *Plant Direct* **4**, 1–11; 10.1002/pld3.257 (2020).

23. Tezuka, T. & Marubashi, W. Hybrid lethality in interspecific hybrids between *Nicotiana tabacum* and *N. suaveolens*: evidence that the Q chromosome causes hybrid lethality based on Q-chromosome-specific DNA markers. *Theor. Appl. Genet.* **112**, 1172–1178 (2006).
24. Tezuka, T., Kuboyama, T., Matsuda, T. & Marubashi, W. Seven of eight species in *Nicotiana* section *Suaveolentes* have common factors leading to hybrid lethality in crosses with *Nicotiana tabacum*. *Ann. Bot.* **106**, 267–276 (2010).
25. Tezuka, T., Matsuo, C., Iizuka, T., Oda, M. & Marubashi, W. Identification of *Nicotiana tabacum* linkage group corresponding to the Q chromosome gene(s) involved in hybrid lethality. *PLoS One* **7**, e37822; 10.1371/journal.pone.0037822 (2012).
26. Goodspeed, T. H. *The genus Nicotiana* (Chronica Botanica Company, 1954).
27. Merxmuller, H. & Buttler, K. P. *Nicotiana* in der afrikanischen Namibein pflanzengeographisches und phylogenetisches Ratsel. *Mitt. Bot. Staatssamml. Münch.* **12**, 91–104 (1975).
28. Purdie, R. W., Symon, D. E. & Haegi, L. *Flora of Australia* (ed. George, A. S.) 1–208 (Australian Government Publishing Service, 1982).
