# Supplementary material for: Two Nicotiana occidentalis accessions enable gene identification for Type II hybrid lethality by the cross to N. sylvestris
Source: Sci Rep. 2021 Aug 24;11:17093. doi: 10.1038/s41598-021-96482-6 (PMC8384851; doi:10.1038/s41598-021-96482-6)
Supplement: Supplementary file 2 — Supplementary Figures. [file 41598_2021_96482_MOESM2_ESM.pdf]

Supplementary Figures

Two *Nicotiana occidentalis* accessions enable gene identification for Type II hybrid lethality by the cross to *N. sylvestris*

Kenji Kawaguchi[1,§], Yuichiro Ohya[1], Maho Maekawa[2], Takahiro Iizuka[1], Akira Hasegawa[2], Kumpei Shiragaki[1], Hai He[1], Masayuki Oda[1,3], Toshinobu Morikawa[1,3], Shuji Yokoi[1,3,4], Takahiro Tezuka[1,3,*]

[1]Graduate School of Life and Environmental Sciences, Osaka Prefecture University, Sakai, Osaka 599-8531, Japan

[2]School of Life and Environmental Sciences, Osaka Prefecture University, Sakai, Osaka 599-8531, Japan

[3]Education and Research Field, College of Life, Environment, and Advanced Sciences, Osaka Prefecture University, Sakai, Osaka 599-8531, Japan

[4]Bioeconomy Research Institute, Research Center for the 21st Century, Osaka Prefecture University, Sakai, Osaka 599-8531, Japan

[§]Present address: NARO Hokkaido Agricultural Research Center, Memuro Research Station, 9-4 Shinsei-minami, Memuro, Kasai, Hokkaido 082-0081, Japan

*Corresponding author: Takahiro Tezuka

Email: tezuka@plant.osakafu-u.ac.jp

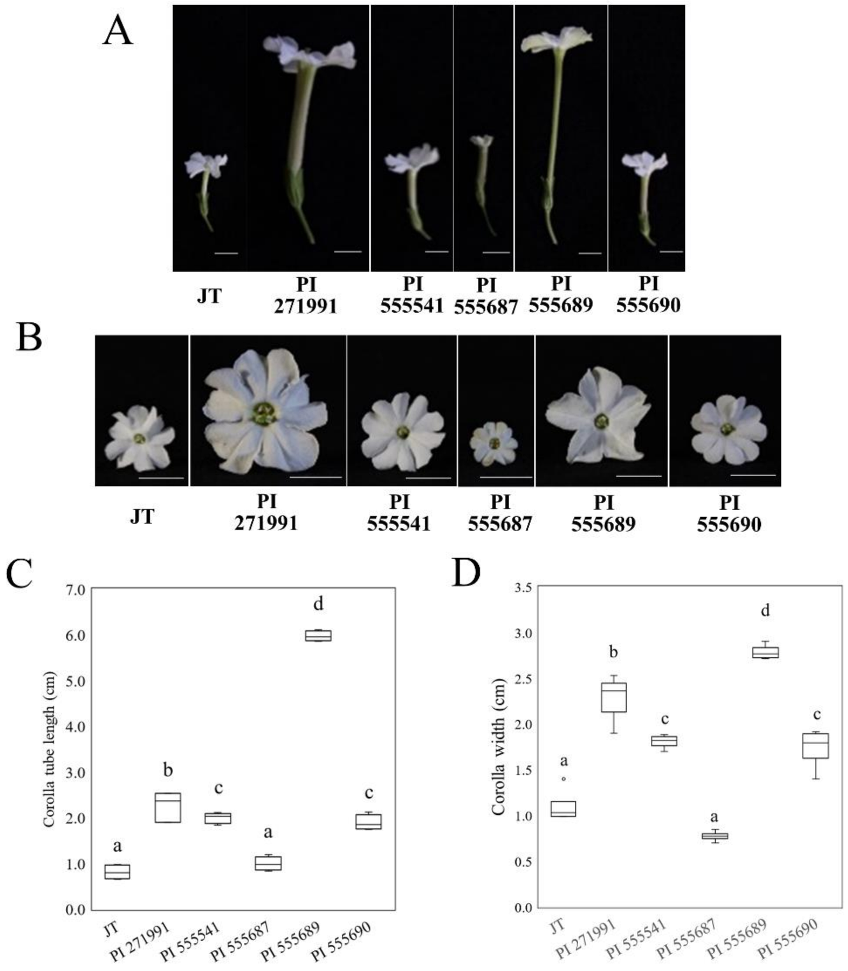

Supplementary Fig. S1. Comparison among *N. occidentalis* JT and five *Nicotiana* spp. accessions in corolla tube length and corolla limb width. (A-B) Appearance of flowers. Scale bars = 1 cm. (C-D) Corolla tube length and corolla limb width in each accession (n = 3–4). Different letters in each accession show significant difference by Tukey–Kramer test at a 5%.

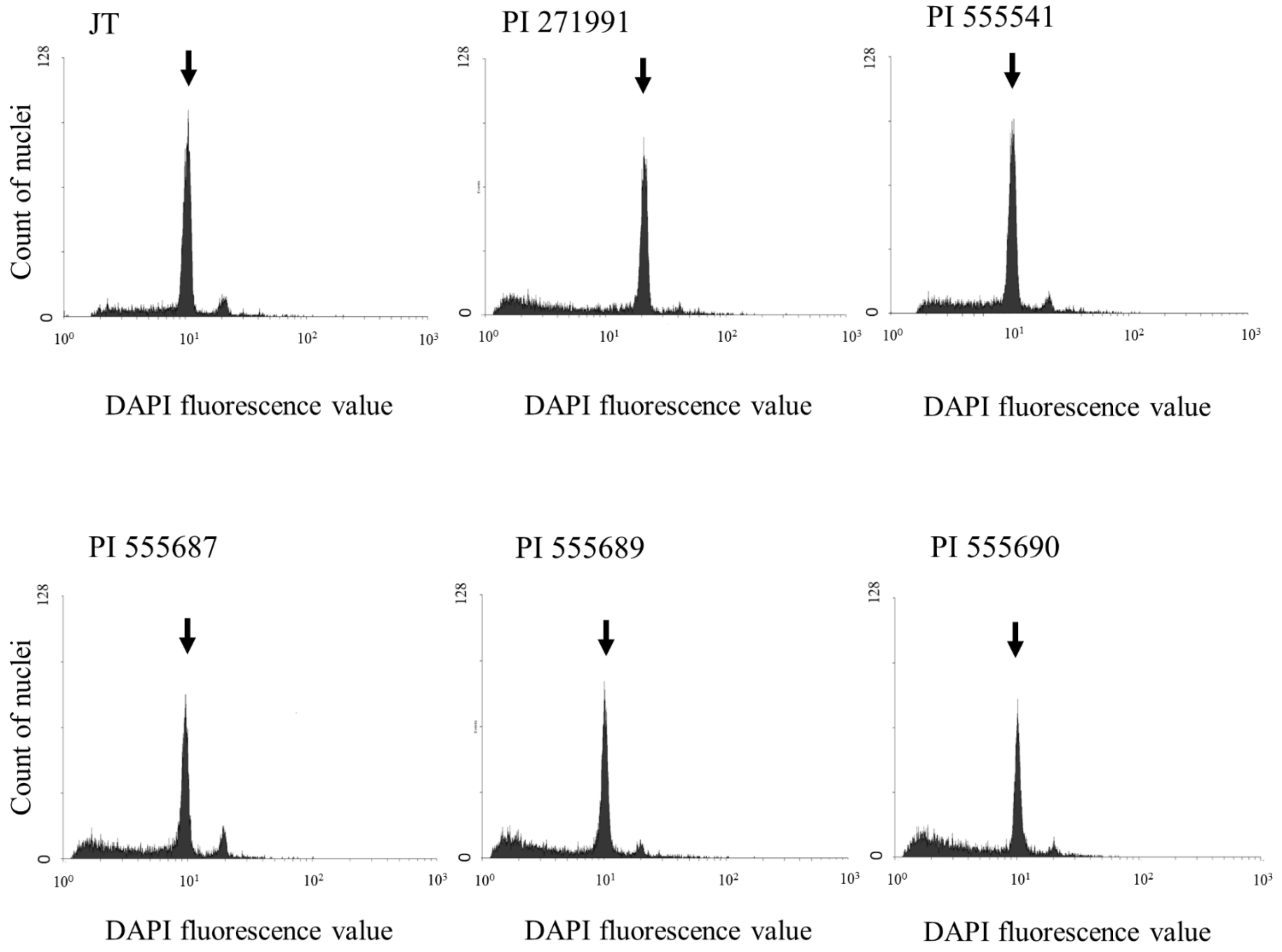

Supplementary Fig. S2. Nuclear DNA content in *N. occidentalis* JT and five *Nicotiana* spp. accessions determined by flow cytometry. Arrows indicate the peaks corresponding to G_1 phase.

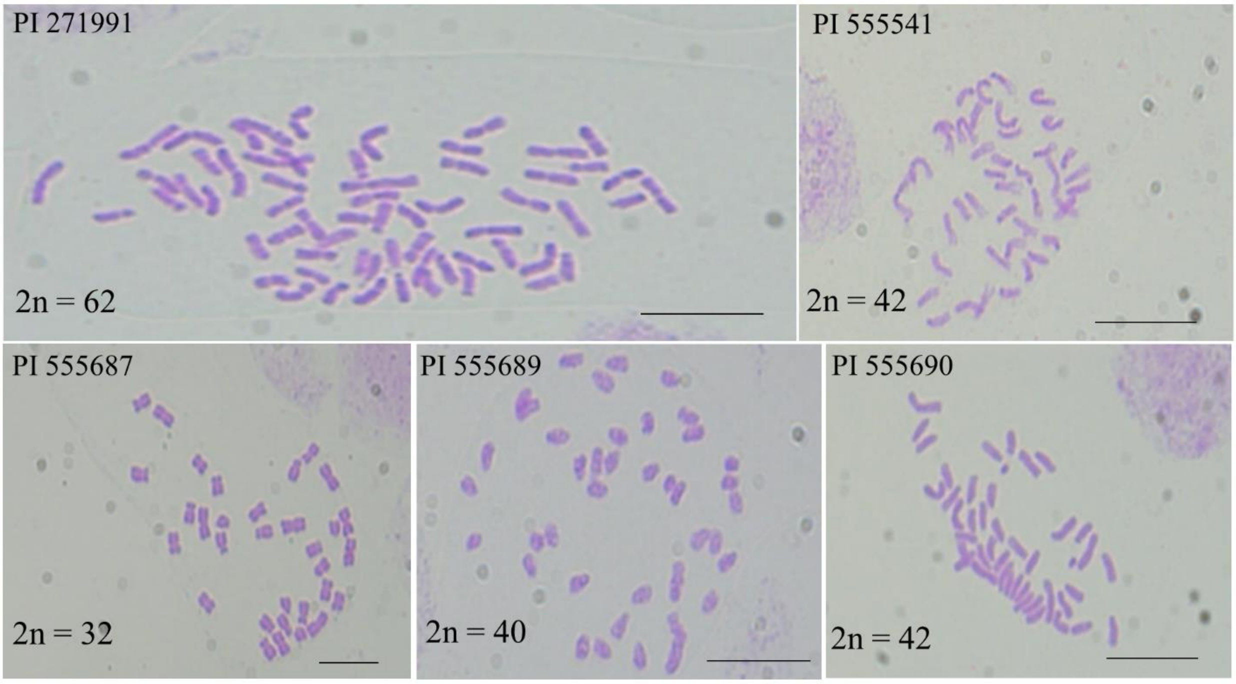

Supplementary Fig. S3. Image of a root tip cell of five *Nicotiana* spp. accessions showing the number of chromosomes. Scale bar = 10 µm.

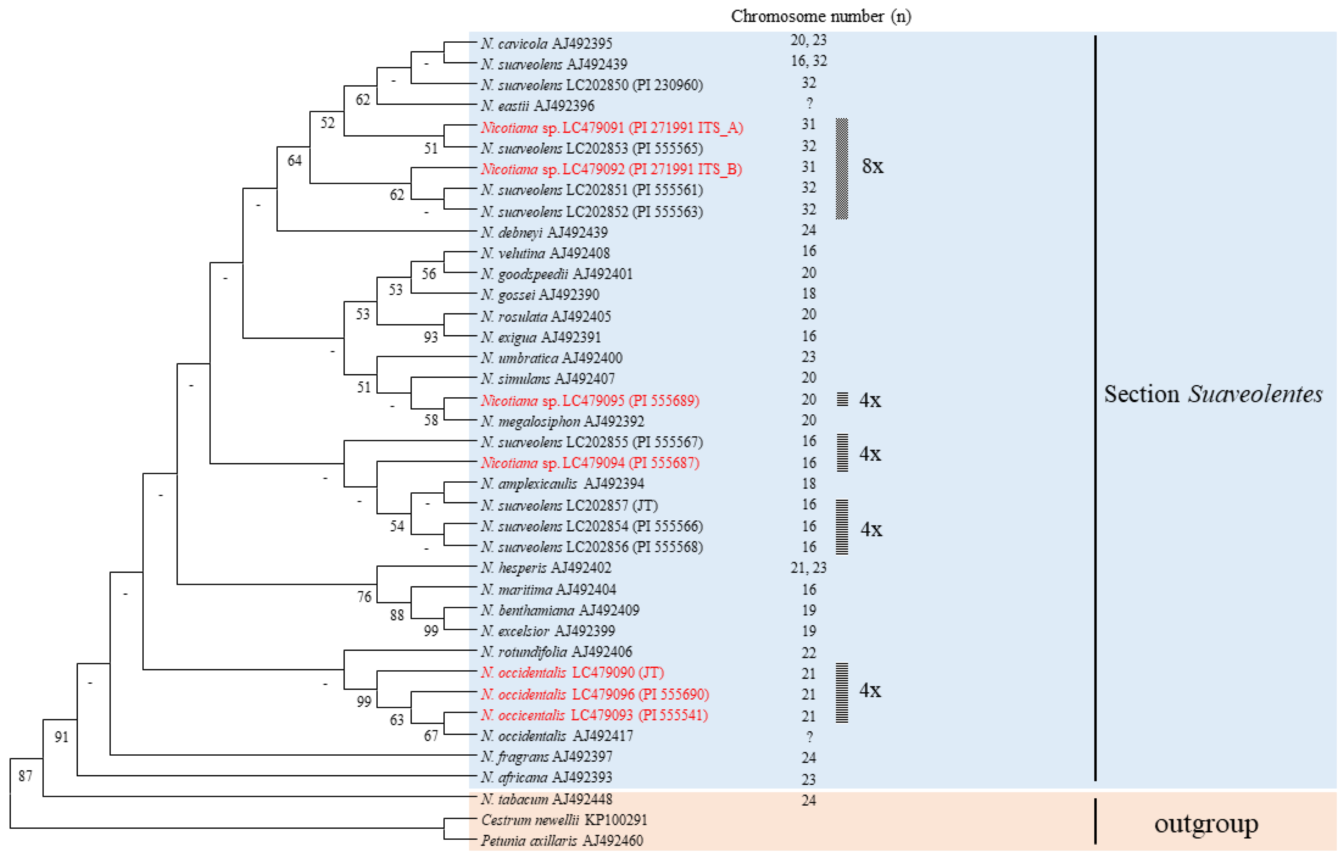

Supplementary Fig. S4. Phylogenetic tree from the maximum likelihood analysis of section *Suaveolentes* using ITS sequence. Species or accessions in red and black represent samples sequenced in this study and referenced samples from Chase et al.[8] and He et al.[17], respectively. Bootstrap percentages are indicated below each branch (any clade with a hyphen has BP <50). Chromosome numbers are referenced from Goodspeed[26], Merxmueller and Buttler[27], Purdie et al.[28], Japan Tobacco Inc.[16], Chase et al.[8], He et al.[17] and the present study.

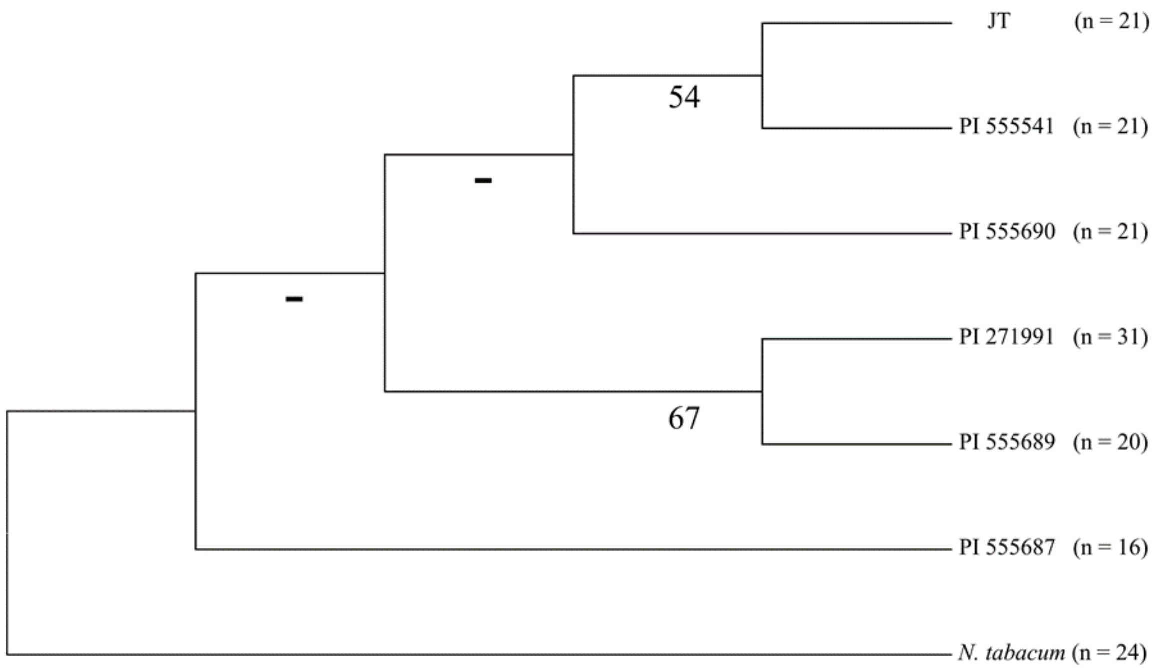

Supplementary Fig. S5. Phylogenetic tree from Maximum parsimony analysis of *N. occidentalis* JT and five *Nicotiana* spp. accessions using SSR markers. Bootstrap percentages are indicated below each branch (any clade with a hyphen has BP <50). Chromosome numbers are referenced from Japan Tobacco Inc.[16] and the present study.

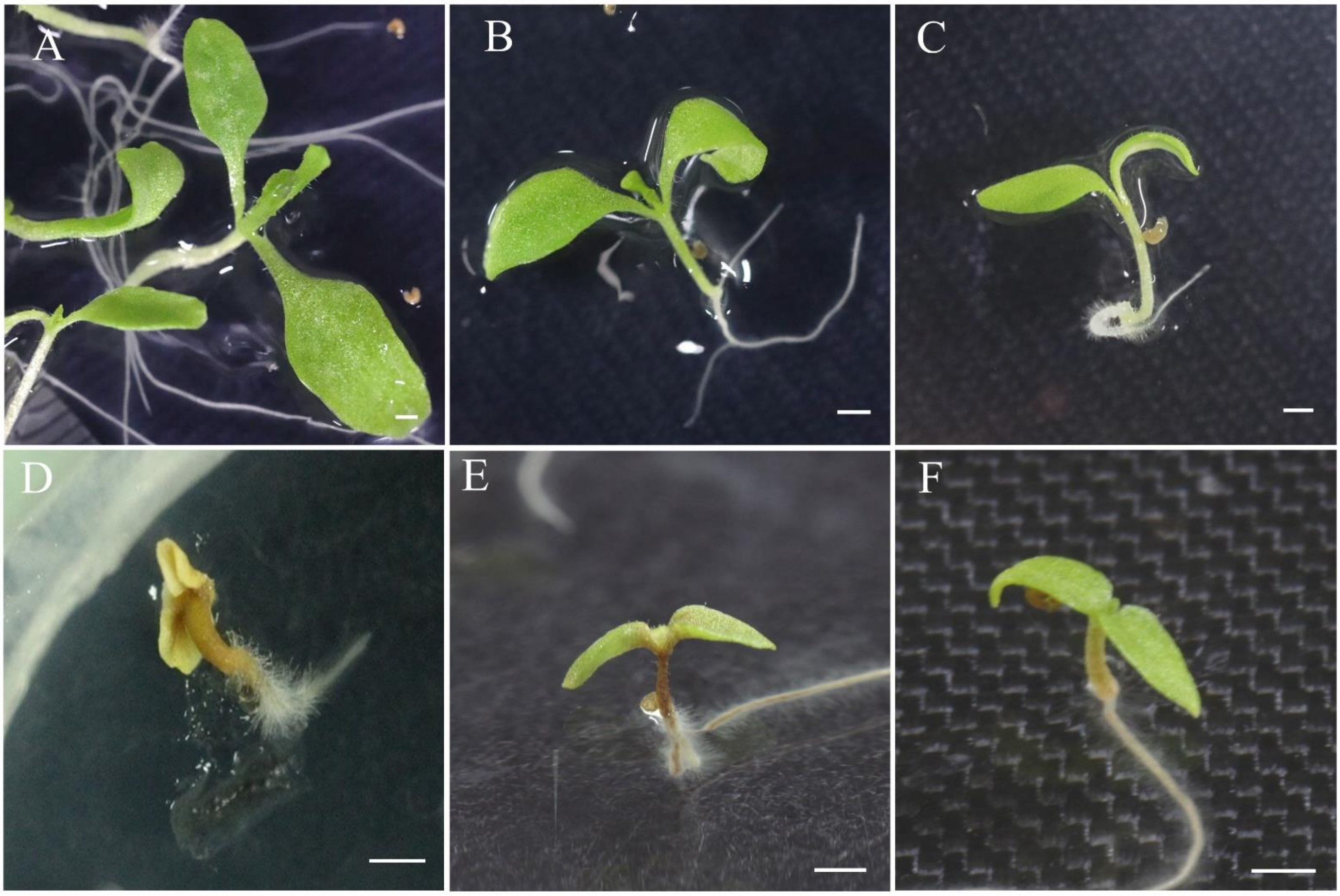

Supplementary Fig. S6. Observation of the characteristic early symptoms of hybrid lethality in hybrid seedlings between each accession of *Nicotiana* spp. and *N. tabacum* at 25°C. Browning of hypocotyl and roots (Type II lethality) were observed in all hybrid seedlings (D-F). (A) PI 271991 at 6 DAG; (B) PI 555687 at 7 DAG; (C) PI 555689 at 2 DAG; (D) Hybrid of PI 271991 at 6 DAG; (E) Hybrid of PI 555687 at 7 DAG; (F) Hybrid of PI 555689 at 2 DAG. Scale bars = 1 mm.

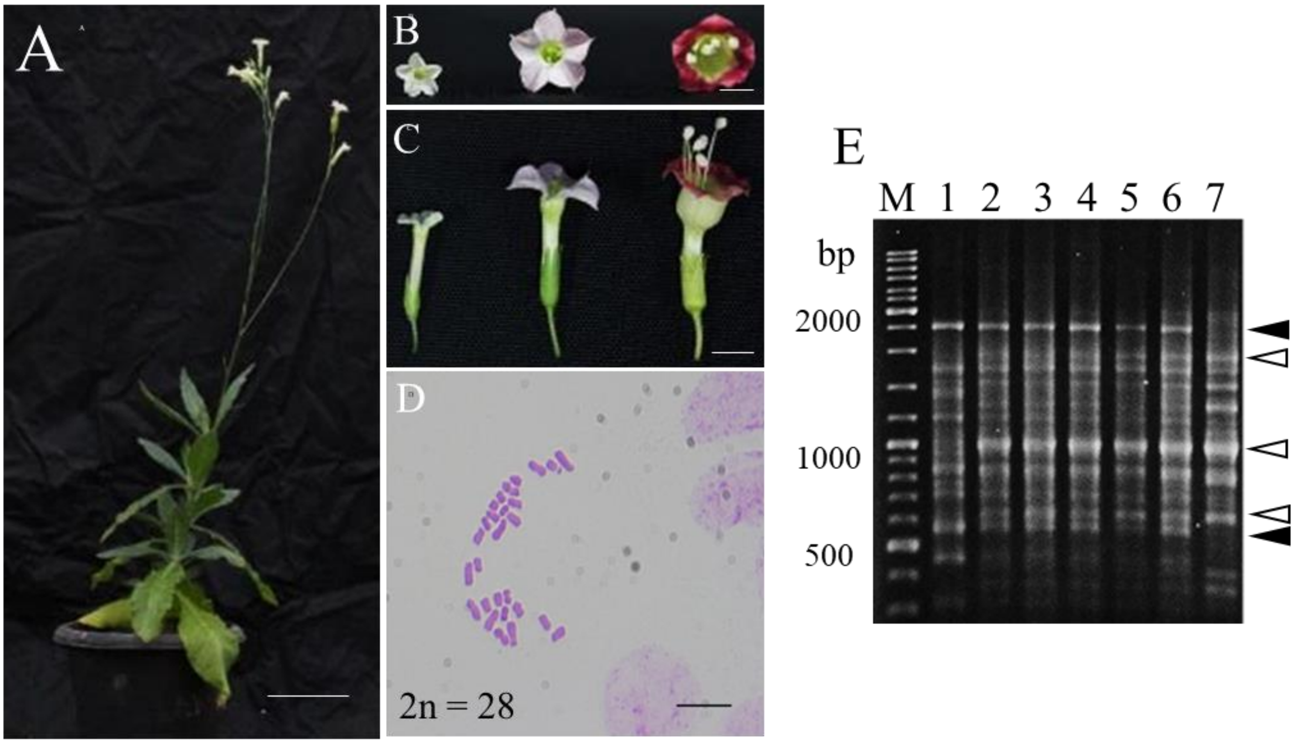

Supplementary Fig. S7. Hybrids from the cross *Nicotiana* sp. PI 555687 × *N. tomentosiformis*. (A) Shape of a hybrid plant that has grown to maturity and flowered. (B-C) Flowers of PI 555687, a hybrid plant and *N. tomentosiformis* (left to right). (D) Image of a root tip cell of hybrid plant showing the number of chromosomes. Scale bars = 10 cm (A), 1 cm (B-C) and 10 µm (D). (E) Confirmation of hybrid formation by RAPD analysis. M, DNA markers (GeneRuler DNA ladder mix). Lane 1, PI 555541; lanes 2–6, hybrid plants; lane 7, *N. tomentosiformis*. Both bands specific to PI 555687 (closed triangles) and those specific to *N. tomentosiformis* were detected in hybrids (open triangles).

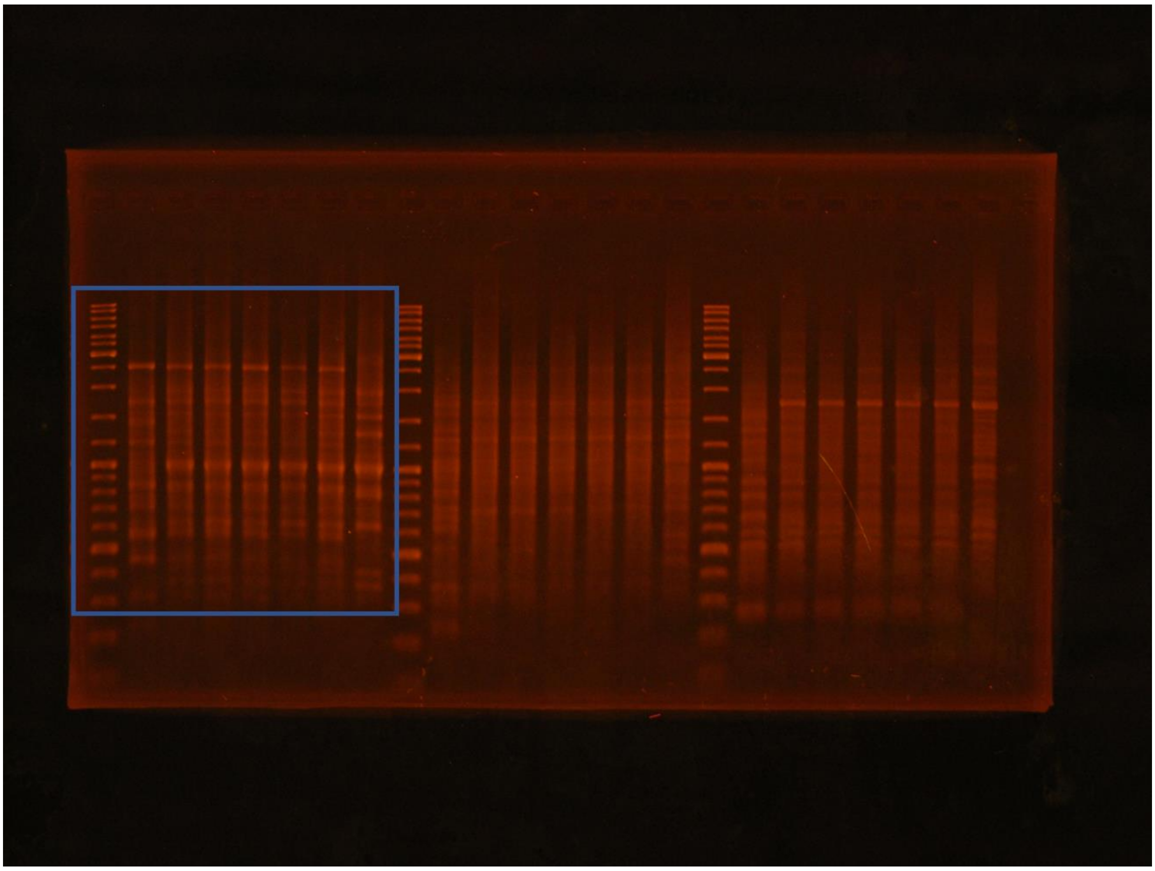

Supplementary Fig. S8. Agarose gel image related to Fig. S7E. Blue frame shows cropped area for Fig. S7E.

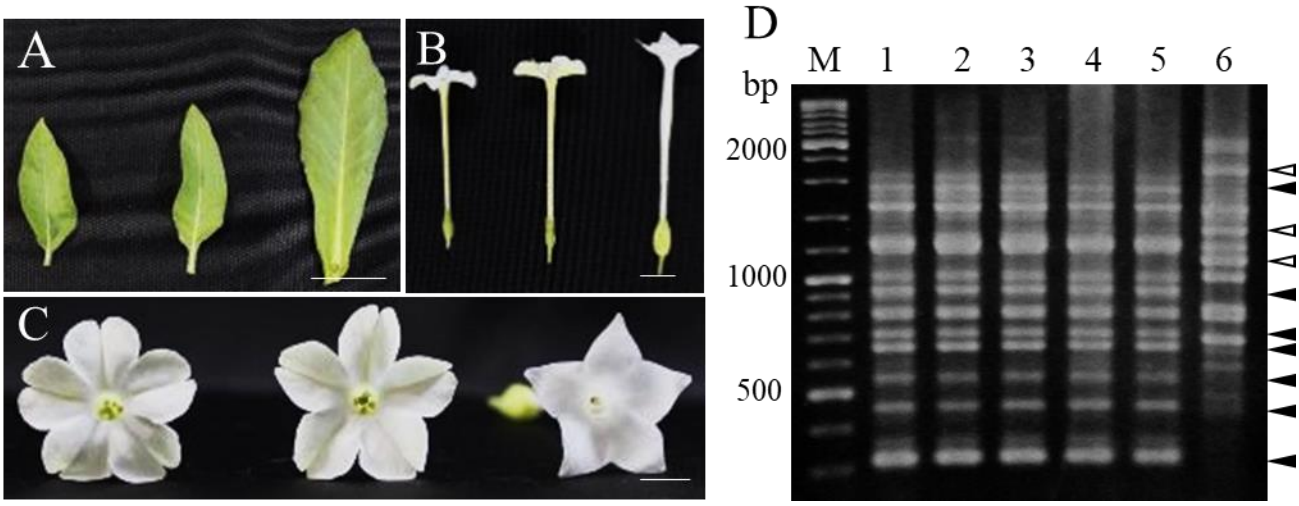

Supplementary Fig. S9. Hybridity analysis of plants derived from the cross *Nicotiana* sp. PI 555689 × *N. sylvestris*. (A) Leaves of PI 555689, a plant from the cross PI 555689 × *N. sylvestris* and *N. sylvestris* (left to right). (B-C) Flowers of PI 555689, a plant from the cross PI 555689 × *N. sylvestris* and *N. sylvestris* (left to right). Scale bars = 5 cm (A), 1 cm (B-C). (D) Analysis of hybridity by RAPD analysis. M, DNA markers (GeneRuler DNA ladder mix). Lane 1, PI 555689; lanes 2–5, plants from the cross PI 555689 × *N. sylvestris*; lane 6, *N. sylvestris*. Bands specific to PI 555689 (closed triangles) were detected but those specific to *N. sylvestris* (open triangles) were not detected in plants from the cross PI 555689 × *N. sylvestris*.

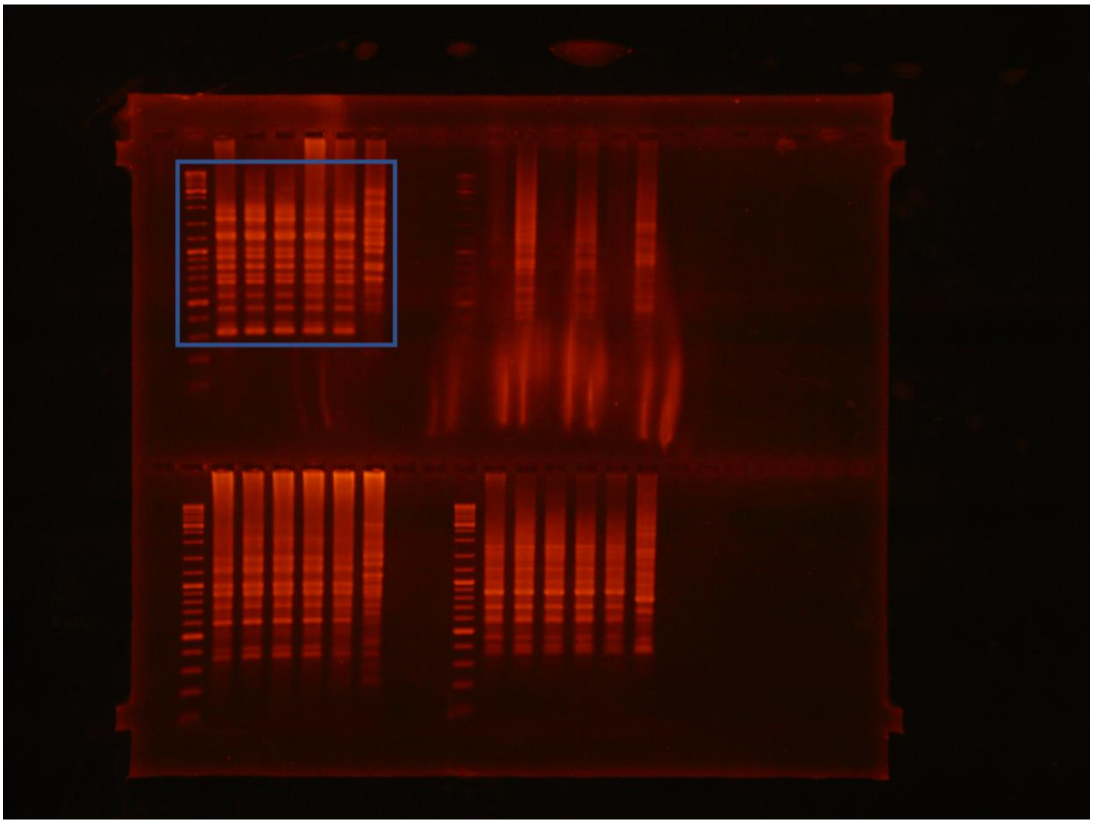

Supplementary Fig. S10. Agarose gel image related to Fig. S9D. Blue frame shows cropped area for Fig. S9D.

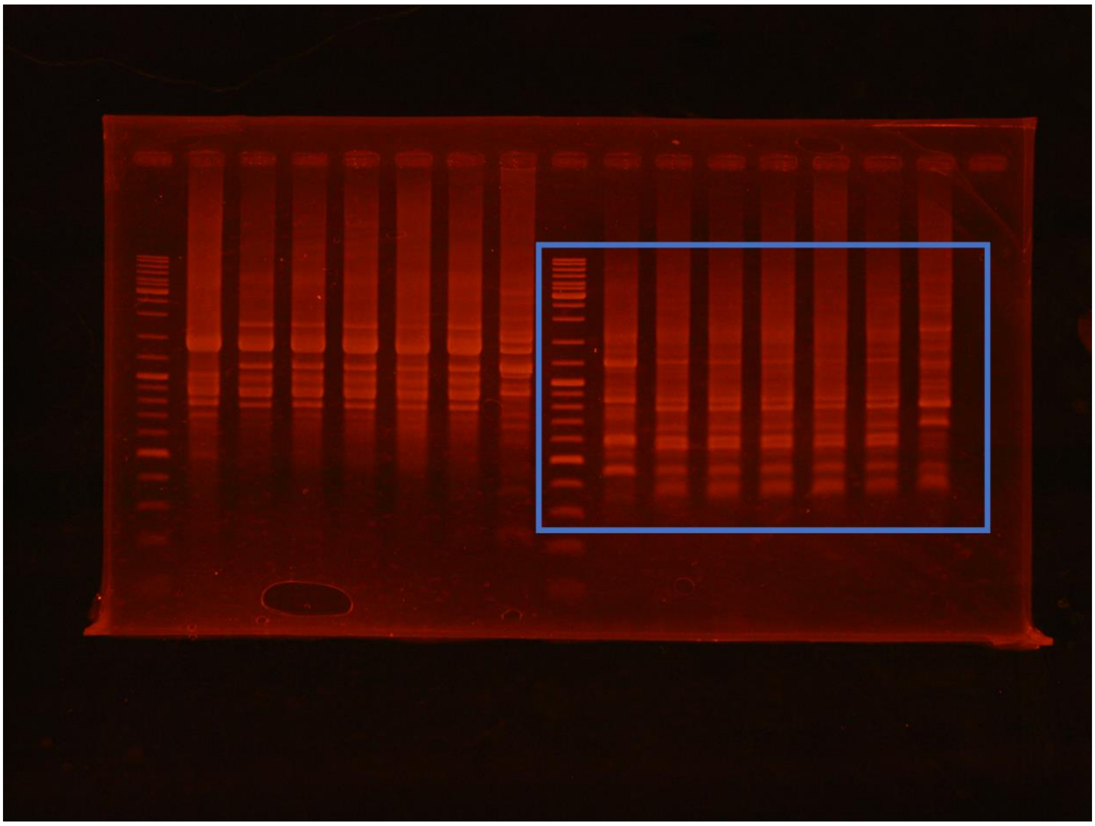

Supplementary Fig. S11. Agarose gel image related to Fig. 2F. Blue frame shows cropped area for Fig. 2F.

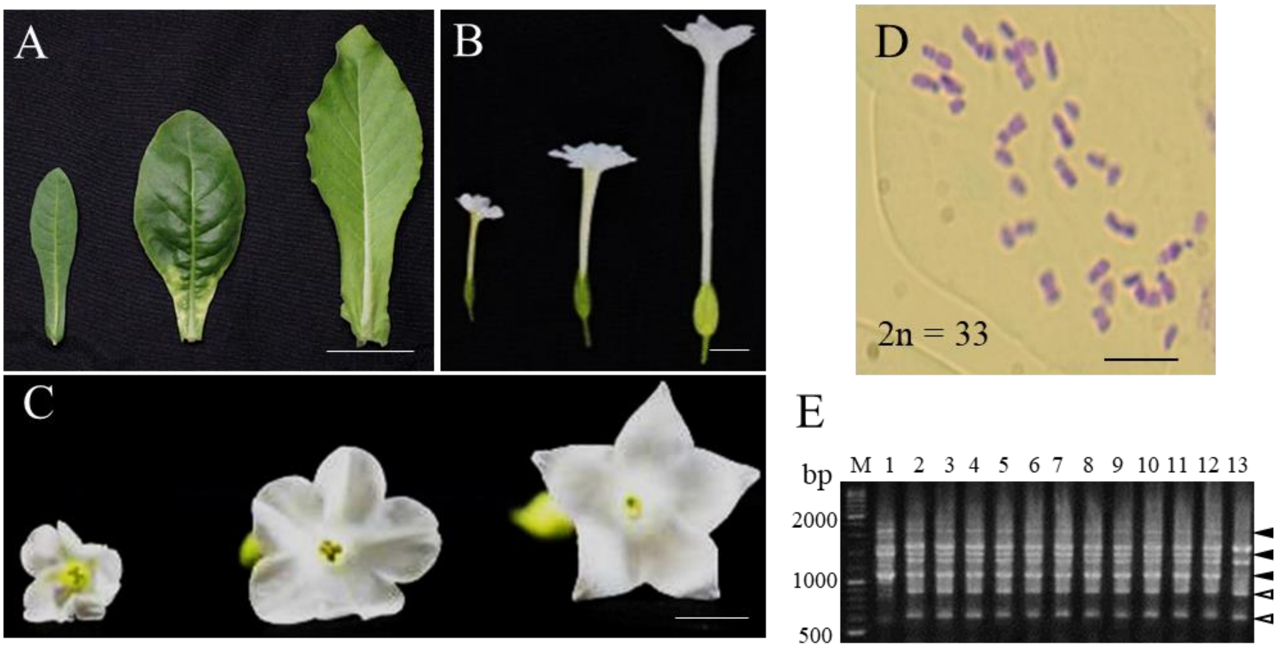

Supplementary Fig. S12. Hybrids from the cross *N. occidentalis* PI 555690 × *N. sylvestris*. (A) Leaves of PI 555690, a hybrid plant and *N. sylvestris* (left to right). (B-C) Flowers of PI 555690, a hybrid plant and *N. sylvestris* (left to right). (D) Image of a root tip cell of hybrid plant showing the number of chromosomes. Scale bars = 5 cm (A), 1 cm (B-C) and 10µm (D). (E) Confirmation of hybrid formation by RAPD analysis. M, DNA size marker (GeneRuler DNA ladder mix). Lane 1, PI 555690; lanes 2–12, hybrid plants; lane 13, *N. sylvestris*. Both bands specific to PI 555690 (closed triangles) and those specific to *N. sylvestris* (open triangles) were detected in hybrids.

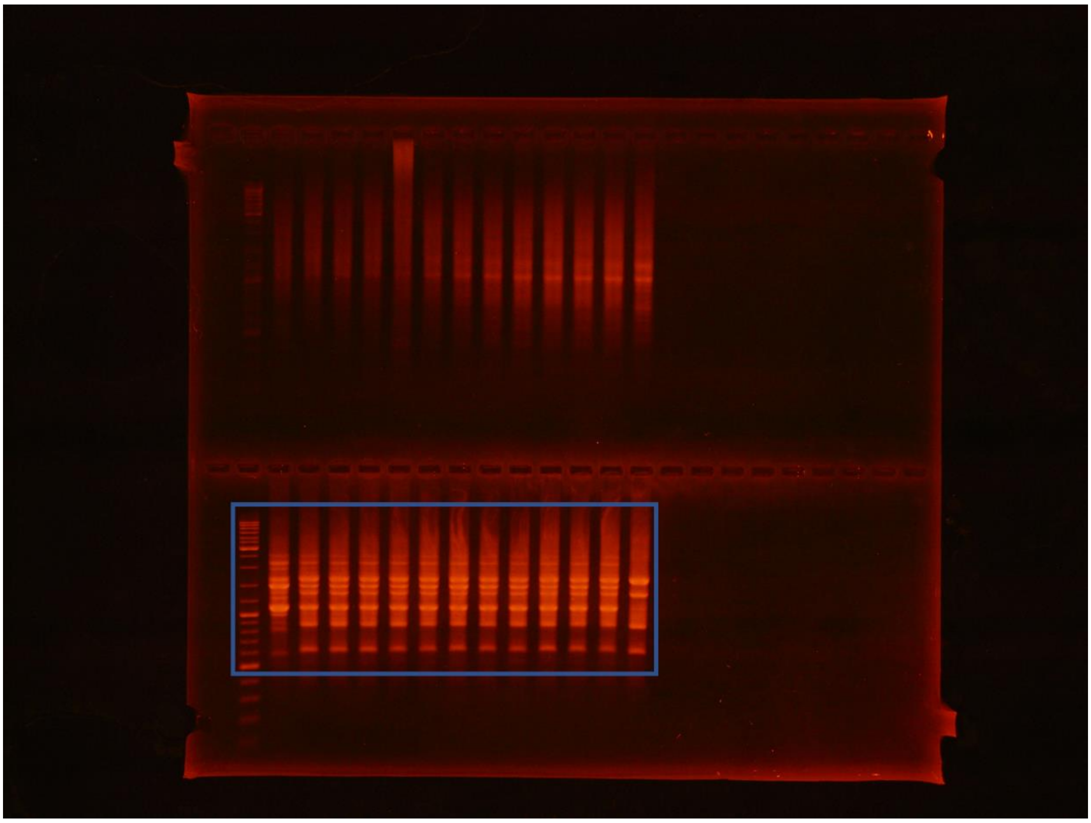

Supplementary Fig. S13. Agarose gel image related to Fig. S12E. Blue frame shows cropped area for Fig. S12E.
